# Sodium Butyrate Promotes Reassembly of Tight Junctions in Caco-2 Monolayers Involving Inhibition of MLCK/MLC2 Pathway and Phosphorylation of PKCβ2

**DOI:** 10.3390/ijms17101696

**Published:** 2016-10-10

**Authors:** Wei Miao, Xiujuan Wu, Kang Wang, Wenjing Wang, Yumei Wang, Zhigang Li, Jingjing Liu, Li Li, Luying Peng

**Affiliations:** 1 Key Laboratory of Arrhythmias, Ministry of Education, East Hospital, Tongji University School of Medicine, Shanghai 200120, China; wikimiao@126.com (W.M.); wuxiujuan1992@163.com (X.W.); tongji_wk@163.com (K.W.); 2015vivian_wang@tongji.edu.cn (W.W.); 14wang_yumei@tongji.edu.cn (Y.W.); happylag5@163.com (Z.L.); ljjdaisywin@163.com (J.L.); 2 Research Center for Translational Medicine, Shanghai East Hospital, Tongji University School of Medicine, Shanghai 200120, China; 3 Department of Pathology and Pathophysiology, Tongji University School of Medicine, Shanghai 200092, China

**Keywords:** butyrate, tight junction, Caco-2, myosin light chain kinase (MLCK), myosin II regulatory light chain (MLC2), (protein kinase C β) PKCβ

## Abstract

As a physiological small molecular product from the microbial fermentation of dietary fibers, butyrate plays an important role in maintaining intestinal health. Our previous works have proved that the effect of sodium butyrate (NaB) on the intestinal barrier function is mediated by activation of AMP-activated protein kinase (AMPK). However, the detailed pathway involved remains unknown. Using the calcium switch assay in the Caco-2 cell monolayer model, we found here that NaB activated AMPK mainly by increasing the calcium level, but not the ATP concentration, via promoting store-operated calcium entry (SOCE). Upon the activation of AMPK, NaB promoted the reassembly of tight junctions (TJs) based on reducing the phosphorylation of myosin II regulatory light chain (MLC2) at Ser19 and increasing phosphorylation of protein kinase C β2 (PKCβ2) at Ser660. Inhibiting (protein kinase C β) PKCβ blocked the reassembly of TJs induced by NaB in the barrier monolayer model. These results indicated that NaB could activate the calcium/calmodulin-dependent protein kinase kinase β (CaMKKβ) pathway to mediate AMPK phosphorylating, which then inhibited the phosphorylation of MLC2 and promoted the phosphorylation of PKCβ2, respectively, so that the downstream molecules of AMPK coordinately contributed to the reassembly of TJs in the Caco-2 barrier model. These results suggested a potential mechanism of butyrate for intestine homeostasis and protection.

## 1. Introduction

Butyric acid, sourced from degradable fiber and degradable starch, is one of the major short-chain fatty acids (SCFA) derived from the fermentation of bacterial carbohydrates [1,2]. Mammalian gastrointestinal epithelia can receive and utilize butyrate as a signaling molecule to regulate proliferation, apoptosis and differentiation to adapt the growth of the bacterial community [3,4]. Butyrate also plays a role as a histone deacetylase inhibitor (HDACi) which regulates gene expressions in cells [5,6]. Our previous research showed that butyrate might improve the barrier function of gastrointestinal epithelia by increasing the transepithelial electrical resistance (TER) of the Caco-2 monolayer when treated with 2 mmol/L sodium butyrate [7], and this effect was attributed to the reassembly of the tight junctions (TJs) through activating AMP-activated protein kinase (AMPK). AMPK as a fuel gauge regulates the activities in glucose and fatty acid metabolism [8]. Generally, canonical regulation of AMPK activity involves the level change of adenine nucleotides or calcium ions [9]. The increase in ADP and AMP relative to ATP ratios is a signal to turn on AMPK in its energy-sensing role by promoting the phosphorylation at Thr172 [10,11], and calcium/calmodulin-dependent protein kinase kinase β (CaMKKβ) mediates another activation mechanism of AMPK through reacting to a rise in intracellular Ca^2+^ [12]. Recently, we found that NaB can activate CaMKKβ in ways other than changing the ATP concentration to mediate the activity of AMPK [13], suggesting sodium butyrate (NaB) may regulate the calcium concentration in Caco-2 cells. A previous study has also shown that NaB induces Ca^2+^ release from the endoplasmic reticulum, and then leads to extracellular Ca^2+^ influx in HCT-116 cells [14]. NaB regulates biological activities in cells in two ways: inhibiting histone deacetylase to impact gene expression or activating G-protein coupled receptors such as GPR43 [15] and GPR109A [16]. GPR109A is a main receptor of butyrate in colonic epithelial cells and mediates the protective effect against colitis [17].

Intestinal function relies on the mucosal barrier which is coated by a monolayer of epithelial cells that are impermeable to most hydrophilic solutes in the absence of specific transporters [18]. The paracellular pathway between cells of an intact epithelial cell layer is sealed by TJs and subjacent adherens junctions. TJs limit solute flux along the paracellular pathway and play major roles in the determination of mucosal permeability [19]. Myosin light chain kinase (MLCK) regulates the permeability of TJs by direct and indirect protein-protein interactions [20,21,22], which are important to maintain the structure and functions of TJs [23]. MLCK-dependent myosin II regulatory light chain (MLC2) phosphorylation is an essential intermediate for physiological regulation of TJs [22]. In smooth muscle cells, the binding of calmodulin with Ca^2+^ activates MLCK by removing an autoinhibitory domain from the kinase catalytic site [24], so that the activated MLCK further phosphorylates MLC2 at Ser19 [25]. Evidence has shown that AMPK can phosphorylate MLCK at Ser815 to inactivate MLCK, resulting in a decrease in the phosphorylation level of MLC2 [26].

PKCβ, including PKCβ1 and PKCβ2, belongs to the classic family of PKC and requires cofactor calcium and diacylglycerol to be activated. Previous research shows that PKCβ1 can protect the barrier integrity of Caco-2 cells from exposure to oxidants, and is also required for EGF-mediated protection of the gastrointestinal barrier [27]. Furthermore, the process in which probiotics ameliorate the hydrogen peroxide-induced epithelial barrier disruption needs the presence of PKC [28]. Therefore, PKCβ plays an important part in regulating epithelial barrier integrity, but whether NaB and AMPK relate to this process remains unclear.

In this work, we found that NaB increased the intracellular calcium level to active AMPK via store-operated calcium entry but not GPR109A in Caco-2 cells.Then the activated AMPK further exerted a role to promote the reassembly of TJs through inhibiting MLCK to reduce the phosphorylation level of MLC2 at Ser19 and increase the phosphorylation level of PKCβ2 at Ser660. Expression levels for some components of the TJ complex were not altered by butyrate (data not shown). These results indicated a complex regulation of NaB in the reassembly of TJs in the monolayer cell model.

## 2. Results

### 2.1. Effect of Sodium Butyrate (NaB) on GPR109A and Store-Operated Calcium Entry (SOCE) during Reassembly of Tight Junctions (TJs)

**Figure 1:**
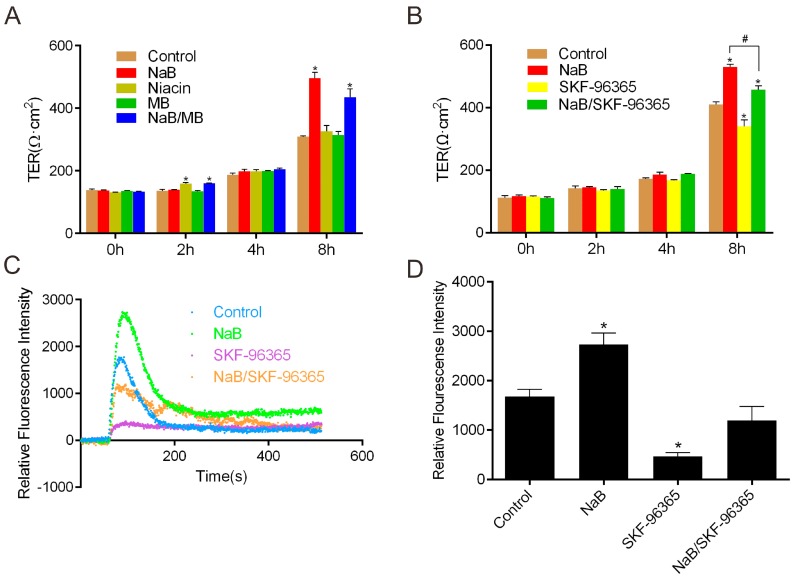
Effect of sodium butyrate (NaB) on GPR109A and store-operated calcium entry. Caco-2 monolayers were incubated in a low-calcium medium for 16 h, and then cell monolayers were switched to Caco-2 culture medium alone or with (**A**) 2 mmol/L of NaB, 2 mmol/L of niacin, 100 nmol/L of mepenzolate bromide (MB) or both NaB and MB, respectively. TERs of the monolayers were detected at the time points of 0, 2, 4 and 8 h; (**B**) 2 mmol/L of NaB, 10 μmol/L of SKF-96365 and NaB/SKF-96365, respectively. TERs of the monolayers were detected at 0, 2, 4 and 8 h; (**C**) Dot plot diagram of [Ca^2+^]i change under the condition of 2 mmol/L of NaB, 5 μmol/L of SKF-96365 and NaB/SKF-96365, respectively; (**D**) Summary of the data in (**C**) which represents the peak values during the examined time interval. Data are expressed as means of relative Fluo-8 fluorescence intensity ± SE (Standard Error), *n* = 3. The asterisks denote a significant difference between chemical-treated groups and controls groups as *p* < 0.05 by two-factor ANOVA. The # symbol denotes a significant difference (*p* < 0.05) between NaB and NaB/SKF-96365. MB—Mepenzolate bromide.

To understand whether the promotion of TJs reassembly by NaB is mediated by butyrate receptor GPR109A, we treated the Caco-2 monolayer using a GPR109A activator (niacin) and inhibitor (mepenzolate bromide) [29] after the calcium-switch assay. We found that either niacin or mepenzolate bromide failed to influence the value of transepithelial electrical resistance (TER) when compared with the effect of NaB on the monolayer (Figure 1A), suggesting the role of NaB on the reassembly of TJs in Caco-2 cells does not depend on the pathway mediated by GPR109A. We then tried to test the effect of NaB on store-operated calcium entry (SOCE). SOCE has been referred to as the phenomenon where the depletion of intracellular Ca^2+^ stores activates Ca^2+^ channels in the plasma membrane as a compensatory mechanism to refill the internal Ca^2+^ store [30], which leads to the result of extracellular Ca^2+^ influx. As one of the major mechanisms for Ca^2+^ entry in non-excitable cells, we hypothesized that SOCE might be involved in the pathway of NaB activating AMPK in the reassembly of TJs. Hereby we performed the function assay for TER change in Caco-2 cells to figure out whether the Ca^2+^ entry is changed in the presence of NaB or the store-operated Ca^2+^ channel (SOCC) inhibitor SKF-96365 [14]. We found that the increase of TER induced by NaB was significantly reduced by SKF-96365 during the reassembly of TJs (Figure 1B). Consistently, the level of intracellular calcium was significantly increased under the condition of NaB, but SKF-96365 blocked the extracellular Ca^2+^ influx and reduced the robust increase in [Ca^2+^]i (intracellular calcium concentration) induced by NaB (Figure 1C,D), suggesting the promotion of TJs reassembly by NaB is mainly attributed to the calcium-mediated activation of AMPK.

### 2.2. Effect of NaB on Myosin Light Chain Kinase (MLCK) and Myosin II Regulatory Light Chain (MLC2) during the Reassembly of TJs

**Figure 2:**
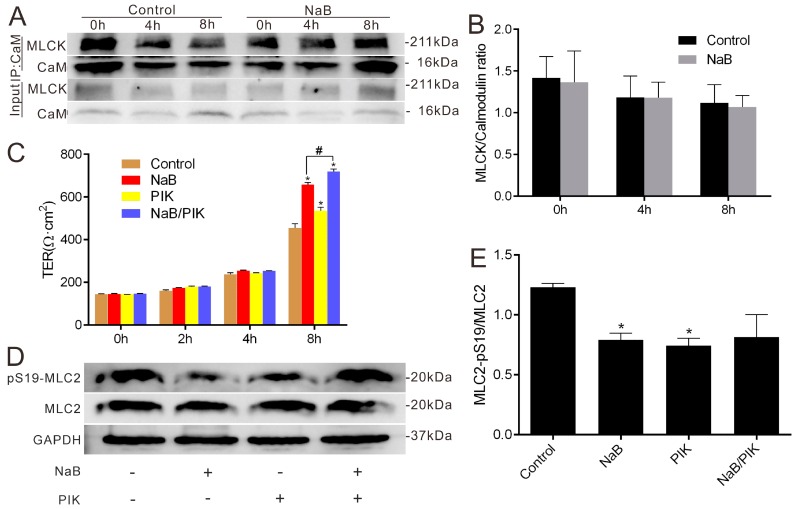
Effects of NaB on the interaction between MLCK and calmodulin as well as phosphorylation levels of MLC2 in Caco-2 cell monolayers. (**A**) After Ca^2+^ switch, Caco-2 cells were cultured in normal Caco-2 medium with or without 2 mmol/L of NaB. Co-Immunoprecipitation (Co-IP) of MLCK and calmodulin was performed at 0, 4 or 8 h, respectively; (**B**) The quantification of MLCK immunoreactive signals by normalized to calmodulin signals in (**A**); (**C**) The change of TERs after Ca^2+^ switch under the condition of 2 mmol/L of NaB, or 250 μmol/L of Permeant inhibitor of MLC kinase (PIK) at 0, 2, 4 and 8 h, respectively; (**D**) Total cell lysates from untreated cells or those treated with 2 mmol/L of NaB or 250 μmol/L of PIK were subjected to immunoblotting for pSer19-MLC2, total MLC2 and GAPDH, respectively; (**E**) MLC2 activity was expressed as the ratio of the phosphorylated form of the MLC2 to total MLC2. Values are means ± SE, *n* = 3. The asterisks denote a significant difference between chemical-treated groups and controls as *p* < 0.05 by two-factor ANOVA. The # symbol denotes a significant difference (*p* < 0.05) between NaB and NaB/PIK. PIK-Permeant inhibitor of MLC kinase.

We hypothesized that MLCK may mediate the effect of NaB on the reassembly of TJs after the calcium switch since the activation of MLCK may involve binding with Ca^2+^-calmodulin, so we tested the association between MLCK and calmodulin. When simulated with NaB for 4 and 8 h, the interaction between MLCK and calmodulin did not change in the process of reassembly (Figure 2A,B). However, treatment with the MLCK-specific inhibitor permeant inhibitor of MLC kinase (PIK) (250 μmol/L) [25] significantly increased the TER of the Caco-2 monolayer after the calcium switch. After treatment with NaB and PIK, the monolayer resistance increased significantly compared with the stimulation of NaB alone (Figure 2C). Generally, the activated MLCK phosphorylates the MLC2 at Ser19 for activation, so we further examined the phosphorylation status of MLC2 at Ser19 under the treatment of NaB or PIK. Similar to the effect of PIK, NaB could also significantly decrease the MLC2 phosphorylation level (Figure 2D,E). These results suggest that the regulation of NaB on the reassembly of TJs depends on the phosphorylation level reduction of MLC2 mediated by MLCK inhibition.

### 2.3. Effect of NaB on the Phosphorylation of Protein Kinase C β (PKCβ) during Reassembly of TJs

**Figure 3:**
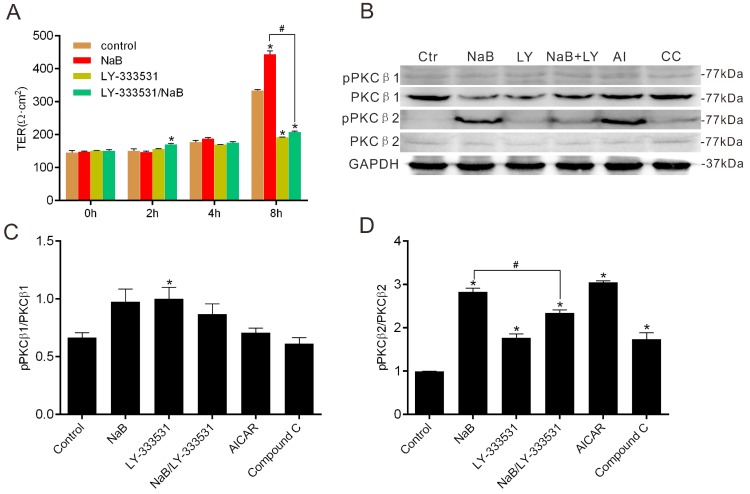
Effects of NaB on PKCβ. (**A**) The change of TERs after Ca^2+^ switch under different conditions (medium alone, or with 2 mmol/L of NaB, or with 5 μmol/L of LY-333531 or LY-333531/NaB) for 0, 2, 4 and 8 h; (**B**) Western blotting at indicated conditions: medium alone; 2 mmol/L of NaB; 5 μmol/L of LY-333531; 1 mmol/L of AICAR; 10 μmol/L of Compound C; (**C**) The ratio of phosphorylated PKCβ1 to total PKCβ1 was quantified in (**B**); (**D**) The ratio of phosphorylated PKCβ2 to total PKCβ2 was quantified in (**B**). Data represent mean ± SE, *n* = 3. The asterisks denote a significant difference between chemical-treated groups and control group as *p* < 0.05 by two-factor ANOVA. The # symbol denotes a significant difference between indicated groups, *p* < 0.05. Ctr—control; LY—LY-333531; AI—AICAR; CC—Compound C.

To explore the role of PKCβ in NaB-treated Caco-2 cells during the reassembly of TJs, we firstly performed the barrier function assay in the presence of the PKCβ inhibitor. We found that PKCβ inhibition caused a significant decrease of the TER of Caco-2 monolayers, even under the condition of NaB (Figure 3A). PKCβ activation generally depends on the autophosphorylation of its isoforms, occurring at serine 660 of PKCβ2 or at threonine 642 of PKCβ1 [31]. Therefore, the phosphorylation status of PKCβ could reflect the activity of two PKCβ isoforms. PKCβ inhibited by LY-333531 [32] obviously prevented the recovery of TERs induced by NaB (Figure 3A). On the other hand, NaB coincidentally increased the phosphorylation level of PKCβ2, but was blocked by the PKCβ inhibitor (Figure 3B–D), consistent with the changes of TER in the Caco-2 monolayer. Moreover, the AMPK activator AICAR showed an increase of phosphorylation of PKCβ2 as the effect of NaB on Caco-2 cells, but the AMPK inhibitor Compound C repressed the phosphorylation (Figure 3B,D). These results indicate that the enhancement of NaB on the barrier function of the Caco-2 monolayer is also involved in the AMPK-mediated activation of PKCβ2.

### 2.4. The Relationship among MLC2, PKCβ2 and AMPK during TJs Reassembly Induced by NaB

**Figure 4:**
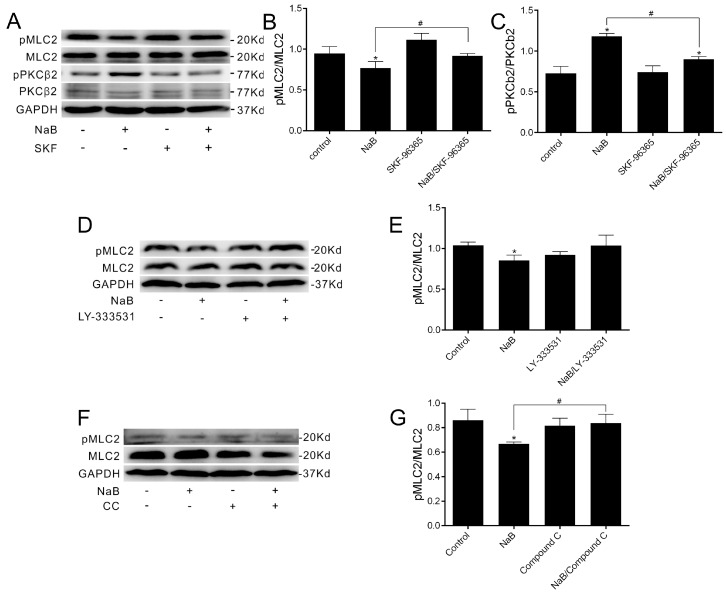
The phosphorylation change of MLC2 and PKCβ2 during TJs assembly induced by NaB. (**A**) Western blotting was performed in the condition of medium alone, 2 mmol/L of NaB and 10 umol/L of SKF-96365; (**B**,**C**) The ratio of phosphorylated MLC2 to MLC2 or phosphorylated PKCβ2 to PKCβ2 in (**A**); (**D**) Western blotting was performed in the condition of medium alone, 2 mmol/L of NaB and 5 μmol/L of LY-333531; (**E**) The ratio of phosphorylated MLC2 to MLC2 in (**D**); (**F**) Western blotting was performed in the condition of medium alone, 2 mmol/L of NaB and 10 μmol/L of Compound C; (**G**) The ratio of phosphorylated MLC2 to MLC2 in (**F**). All experiments were performed at 8 h after calcium switch. Data represent mean ± SE, *n* = 3. The asterisks denote a significant difference between chemical-treated groups and control group as *p* < 0.05 by two-factor ANOVA. The # symbol denotes a significant difference between indicated groups, *p* < 0.05. CC—Compound C.

To further confirm the effect of NaB on the reassembly of TJs mediated by SOCE-induced AMPK activation and the involvement of MLC2 and PKCβ2, immunoblotting was performed to examine the phosphorylation status of pMLC2 and pPKCβ2 in the presence of the SOCE inhibitor at 8 h after the calcium switch. By blocking the calcium influx, the changes of both pMLC and pPKCβ2 induced by NaB were abolished (Figure 4A–C), suggesting the increase of intracellular calcium trigged by butyrate mediates the AMPK/MLC2/PKCβ pathway to promote the reassembly of TJs in Caco-2 cells. Furthermore, we observed the relationship between PKCβ2 and MLC2 in the reassembly of TJs, and found no significant difference in the pMLC2 level treated with NaB or the NaB/PKCβ inhibitor (Figure 4D,E), indicating PKCβ2 may not be involved in the change of the pMLC2 level induced by NaB. When treated with the AMPK inhibitor (Figure 4F,G), the NaB-induced decline of pMLC2 was blocked, further suggesting that NaB-activated AMPK inhibition on the phosphorylation of MLC is essential in the reassembly of TJs.

## 3. Discussion

Butyrate provides energy for intestinal epithelial cells and has various beneficial effects such as inhibition for colon carcinogenesis and regulation in the intestinal barrier [33]. Here, we identified that NaB increased intracellular [Ca^2+^]i in an SOCE-dependent manner to mediate CaMKKβ-induced activation of AMPK and in turn inhibited the MLCK activity, followed by downregulation of the phosphorylation level in MLC2; meanwhile, the activated AMPK also increases the phosphorylation level of PKCβ2 at S660. All of these regulations further facilitate recovery of the barrier function through TJs reassembly in the Caco-2 monolayer. As the central molecule, AMPK may act as a mediator in transferring signals to downstream pathways after being activated by NaB.

Previous work showed that GPR109A, the main cell surface receptor of butyrate, suppresses colonic inflammation and carcinogenesis in the colonic epithelium [17,34]. Our result showed here that GPR109A does not mediate the effect of NaB on TJs reassembly, but SOCE is involved in the role, suggesting the increase of the Ca^2+^ level in cytoplasm depends on the presence of NaB. Since monocarboxylate transporter-1 (MCT-1) mainly transfers Short-Chain Fatty Acid (SCFA) into the colon [35], whether the intracellular butyrate directly induces Ca^2+^ storage to release Ca^2+^ still needs to be elucidated in the future.

The phosphorylation level of MLC2 is controlled by MLCK, and in turn regulates the barrier function. Like the MLCK inhibitor PIK, NaB plays a similar role in repressing the activity of MLCK, showing a decrease in the phosphorylation level of MLC2 and enhancing the TER of Caco-2 cells in reassembly. Also, NaB does not affect the interaction of Ca^2+^-calmodulin with MLCK in the process, consistent with previous results in which the activated AMPK inhibits the activity of MLCK [36]. The further confirmations support that Ca^2+^ and AMPK are involved in the pathway, induced by NaB to MLCK. These observations suggest that in the presence of NaB, the Ca^2+^-calmodulin can not promote the interaction with MLCK for activation, but result in a repression status at the phosphorylation level of its substrate that then might induce TJs reassembly.

As one of the classic subgroup members of the PKC family, PKCβ1 has been shown to mediate the protection of barrier integrity via stabilization of the microtubule and actin cytoskeletons by epidermal growth factor (EGF) or transforming growth factor alpha (TGF-α) signaling [27]. The effect of PKC in reassembly of tight junctions may be mediated by the microtubule or actin cytoskeletons which connect to tight junction proteins. Probiotics ameliorate hydrogen peroxide–induced epithelial barrier disruption in a PKCβ1- and PKCε-dependent manner [28]. Here, we found PKCβ2 is involved in TJs reassembly after being phosphorylated at Ser660 by NaB-mediated AMPK activation in the Caco-2 cell model. This implies a potential way that involves AMPK may exist for activating PKCβ2. When we blocked PKCβ using a specific inhibitor, NaB lost the effect of promoting the barrier function recovery, indicating the key role of PKCβ in epithelial protection induced by butyrate. We also confirm that blocking SOCE could turn over the effects of NaB on both MLC2 and PKCβ2 in TJs reassembly, indicating CaMKKβ-mediated AMPK activation regulates MLC2/PKCβ2 signaling in barrier function recovery. However, the details of the coordinated effects of AMPK with both MLCK and PKCβ2 need to be further elucidated. As one of the activators of PKCβ, diacylglycerol may also mediate the effect of butyrate on PKCβ, which should be considered in our further study.

PKC activation was found to increase the permeability of the endothelial cell barrier [37,38,39,40,41], but other studies showed that the inhibition of PKCβ by a new kind of specific inhibitor promotes vascular endothelial cell barrier permeability [42]. The discrepancy may be due to the differences in the tissue cell types used and the methodologies for detecting barrier function. PKCβ may also exert different influences involving alternative mechanisms in the process of barrier development and recovery. Our conclusion here arises from the epithelial cell model and is limited to the dynamic of TJs reassembly regulated by NaB. On the other hand, Valenzano et al. showed that after Caco-2 cell layers were cultured for 72 h with butyrate, protein expression of claudin-2 decreased 90% and that of claudin-7 increased 376% [43], suggesting butyrate might also regulate the remodeling of the TJs complex along with the differentiation state of Caco-2 cells for maturing the barrier function.

## 4. Materials and Methods

### 4.1. Cell Culture

The human colonic epithelial cell line Caco-2 was purchased from Shanghai Institutes for Cell Resource Center (Shanghai, China). The cells (passage 20–35) were maintained routinely in Corning 75 cm^2^ tissue culture flask at 37 °C with 95% air atmosphere about 95% humidity and 5% CO_2_. The Caco-2 monolayers were grown in Dulbecco’s Modified Eagle’s Medium (DMEM) with high glucose (4500 mg/L) and l-glutamine, supplemented with 10% (*v*/*v*) fetal bovine serum, 100 U/L penicillin-streptomycin, and 1× nonessential amino acids. All of these cell culture reagents were purchased from Life Technology (Carlsbad, CA, USA).

### 4.2. Chemicals

Sodium butyrate, SKF-96365, Pluronic^®^ F-127, Niacin, Mepenzolate bromide and LY-333531 hydrochloride were purchased from Sigma-Aldrich (St. Louis, MO, USA). Compound C and Fluo-8 were purchased from Abcam (Cambridge, UK). Permeant inhibitor of MLC kinase (PIK) was synthetized by Top-peptide (Shanghai, China) as described previously [25].

### 4.3. Calcium Switch Assay

No more than 48 h after fully confluence Caco-2 cell monolayers were washed with Calcium free S-MEM (11380037, Life Technology, Carlsbad, CA, USA) for two times and cultured with Calcium free S-MEM for 16 hand washes with DMEM. Then Caco-2 monolayers either incubated in the Caco-2 culture medium (contain the normal Ca^2+^ concentration ≥1.8 mM) or supplemented with specific chemicals for the indicated time. All the culture medium changed at the same time. Reassembly of TJs and restoration of barrier function were determined at various time points and treatment by measuring the TER.

### 4.4. Measurement of TER

TER assay of Caco-2 monolayers were performed in 12-well Transwell Permeable Supports (Corning, MA, USA) as described in detail previously [39]. Briefly, 5 × 10^6^ Caco-2 cell were seeded with 0.5 mL Caco-2 culture medium in each Transwell insert which bathed in wells of 12-well cell culture plate (Corning, MA, USA) with 1.5 mL Caco-2 culture medium. TER was measured using EVOM2 epithelial voltohmmeter (WPI, Bar Harbor, ME, USA).

### 4.5. Intracellular Ca^*2+*^ Measurements

Ca^2+^-sensitive fluorescent indicator Fluo-8 AM ester (Abcam, Cambridge, UK) was used to measure intracellular Ca^2+^ concentration ([Ca^2+^]i) by an inverted laser scanning confocal microscope (Leica TCS SP5, Wetzlar, Germany). After Caco-2 cells were cultured in S-MEM for 15 h, cells were loaded with 5 μmol/L Fluo-8 AM at 37 °C for 40 min in dark condition, then were rinsed with S-MEM and kept at room temperature (about 25 °C) for 20 min to allow de-esterification of Fluo-8 ester before treated with NaB or SOCE inhibitor SKF-96365 (5 μmol/L) in HBSS (Life Technology, Carlsbad, CA, USA). Laser light was set at 488 nm and emitted fluorescence light was detected through a 514 nm channel. Images of Fluo-8 AM was recorded to analyze fluctuation of [Ca^2+^]i which are presented as ΔF/F0 ratios after background subtraction, where ΔF was the change in fluorescence signal intensity and F0 was calculated by the LAS X (Leica Application Suite X software (Wetzlar, Germany)).

### 4.6. Western Blotting

After the appropriate incubation, the Caco-2 cells were washed with calcium and magnesium free PBS and lysed in RIPA lysis buffer (150 mM sodium chloride, 0.5% sodium deoxycholate, 1.0% Triton X100, 50 mM Tris, 0.1% SDS, pH 8.0) with protease Inhibitor cocktail and phosphatase inhibitor cocktail (Biotool, Houston, TX, USA). The cell lysate was centrifuged at 13,000× *g* for 15 min at 4 °C to yield a clear lysate. Protein concentration was determined by BCA Protein Assay Kit (Takara, Kusatsu, Shiga, Japan). Cell extracts were mixed with a quarter volume of Laemmli sample buffer (5× concentrated, 0.25% Bromophenol blue, 0.5 M dithiothreitol, 50% Glycerol, 10% SDS, 0.25 M Tris-HCl pH 6.8) and heated at 95 °C for 10 min. Equal concentrations of protein (30–50 μg) were separated using 8%–12% SDS-PAGE gel and then were transferred onto Polyvinylidene fluoride (PVDF) blotting membranes (Immobilon-P, Millipore, Temecula, CA, USA). The target proteins on membranes were respectively blotted using primary antibodies of S19 phospho-MLC2 (1:2000), MLC2 (1:1000), (Cell Signaling Technology, Danvers, MA, USA), PKCβ1 (1:500), PKCβ2 (phosphor S660) (1:50,000, Abcam, USA), p-PKCβ1 (Thr641) (1:200), PKCβ2 (1:200, Santa Cruz, CA, USA), and GAPDH (1:2000, Proteintech, Rosemont, IL, USA). The signals were visualized with the SuperSignal West Pico Chemiluminescent Substrate (Thermo Scientific, Waltham, MA, USA).

### 4.7. Immunoprecipitation

After the calcium switch, the cells were washed with Ca^2+^ and Mg^2+^ free PBS and then suspended in IP lysis buffer (150 mM NaCl, 20 mM Tris HCl pH 8, 2 mM EDTA, 1% Triton ×100), Protease Inhibitor Cocktail and Phosphotase Inhibitor Mix (Biotool, Houston, TX, USA). The cell extracts were subjected to centrifugation at 13,000× *g* for 15 min, then incubated with anti-I-afadin or anti-Calmodulin (Abcam, Cambridge, UK) and protein G Plus/Protein A Agarose Suspension beads (EMD Millipore, Billerica, MA, USA) at 4 °C for 4 h. The beads in the bottom of tube were extensively washed with IP lysis buffer for three times, then boiled to elute bound protein in 5× Laemmli buffer for 7 min. The eluted proteins were then subjected to SDS-PAGE, followed by Western blotting with the anti-MLCK (1:7500) and anti-Calmodulin (1:1000) antibody, respectively.

### 4.8. Statistical Analysis

All experiments were repeated at times indicated. Multiple groups data at multi-time point were compared for each treatment with control by two-factor ANOVA and Dunnett’s test for post hoc comparisons. All data presented as mean ± SE. A value of *p* < 0.05 was considered significant in each case. All of statistical analyses were performed using GraphPad Prism software for Windows.

## 5. Conclusions

**Figure 5:**
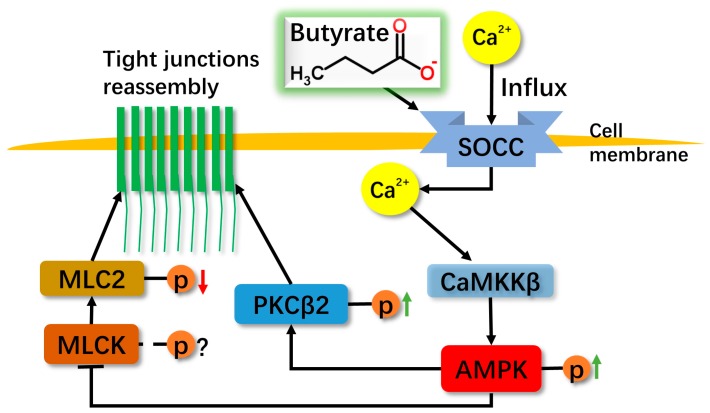
Proposed diagram showing the mechanism of NaB on reassembly of tight junctions in Caco-2 monolayers. NaB appears to activate Store-Operated Ca^2+^ Channel (SOCC) which conducts the Ca^2+^ influx and then activates CaMKKβ/AMPK, resulting in PKCβ2 and MLCK/MLC2 pathways to mediate barrier function recovery. The black arrows indicate active effects, and the line with bar at the end indicates inhibition effect. The red arrow indicates reduction, and the green arrows indicates increase.

In conclusion, NaB induces the influx of extracellular Ca^2+^ through SOCE to activate AMPK mediated by the Ca^2+^/CaMKKβ pathway, and then leads to the reduction of the phosphorylation level of MLC2 which is attributed to the inhibition of MLCK. Meanwhile, the activated AMPK also induces the phosphorylation of PKCβ2. All of the NaB-mediated effects totally promote the recovery of barrier function in the Caco-2 cell monolayer model after the calcium switch (Figure 5), suggesting a complex regulation by butyrate on epithelial protection.
